# Parental Perceived Travel Time to and Reported Use of Food Retailers in Association with School Children’s Dietary Patterns

**DOI:** 10.3390/ijerph16050824

**Published:** 2019-03-07

**Authors:** Mariane de Almeida Alves, Maria Gabriela M. Pinho, Elizabeth Nappi Corrêa, Janaina das Neves, Francisco de Assis Guedes de Vasconcelos

**Affiliations:** 1 Department of Nutrition, Centre of Health Sciences, Federal University of Santa Catarina, Trindade, Florianópolis, Santa Catarina 88040-900, Brazil; marianeaalves@hotmail.com (M.d.A.A.); elizabeth.nappi@ufsc.br (E.N.C.); janaina.neves@ufsc.br (J.d.N.); f.vasconcelos@ufsc.br (F.d.A.G.d.V.); 2 Department of Epidemiology and Biostatistics, Amsterdam Public Health Research Institute, Amsterdam UMC, Vrije Universiteit Amsterdam, F wing, De Boelelaan 1089a, 1081 HV Amsterdam, The Netherlands

**Keywords:** school children, dietary patterns, food environment, perceived travel time, use of food retailers

## Abstract

Considering the association between the neighborhood food environment and individual eating behaviors, this study aimed to assess the association between parents’ reported use of food facilities by their children, and parental perceived travel time to food facilities, with their children’s dietary patterns. Parents reported the use of supermarkets, full-service and fast-food restaurants, and perceived travel time to these food retailers. To assess school children’s food consumption, a previous day dietary recall was applied. Factor analysis was conducted to identify dietary patterns. To test the association between reported use and perceived travel time to food retailers and school children’s dietary patterns, we performed multilevel linear regression analyses. Parents’ reported use of supermarkets was associated with children’s higher score in the “Morning/Evening Meal” pattern. The use of full-service and fast-food restaurants was associated with children’s higher score in the “Fast Food” pattern. Higher parental perceived travel time to full-service and fast-food restaurants was associated with children’s lower score in the “Fast Food” pattern. Although the use of full-service and fast-food restaurants was associated with a less healthy dietary pattern, the perception of living further away from these food retailers may pose a barrier for the use of these facilities.

## 1. Introduction

There is a growing amount of evidence for an association between the neighborhood food environment and individual eating behaviors [1,2]. Considering that food choices are not only determined by individual behaviors, but are also influenced by social and physical environments [3], policies and interventions aimed at improving dietary patterns should take into account the environment in which people live, make food choices, and consume foods [4].

Exposure to the food environment comprises access to many different types of food retailers. The literature shows that supermarkets are an important source of food for many families [5] and living closer to a supermarket is associated with its use [6] and children’s food choices [7]. Higher access to supermarkets is associated with lower consumption of fast food among adults [8] and with improved consumption of fruits and vegetables among children [9]. However, supermarkets are also associated with less desirable food choices such as increased consumption of processed products at the expense of minimally processed foods [10].

Furthermore, a higher proximity from a school or residence to fast-food restaurants is associated with unhealthier diets among students, including lower consumption of fruits and vegetables [11], higher portions of soda [11,12], and higher consumption of fatty foods [12]. Full-service restaurants are also an important component of the food environment that are very often neglected by food environment research [13]. From international literature, a link was observed in the exposure to full-service restaurants and a higher consumption of sugar-sweetened beverages [14], a lower consumption of fruits and vegetables [12,15], and higher calorie intake [16]. From a Brazilian perspective, buffet restaurants serving lunch might be a resource for maintaining a healthy diet outside the home, as these food retailers provide opportunities for a higher adherence to traditional Brazilian cuisine based on rice, beans, fresh meat, roots, and vegetables [17].

Exposure to food retailers can be assessed using different measures, tools, and methodological procedures, including metrics based on geographic information systems [1], which can be used, for instance, to objectively quantify the availability of food retailers in a neighborhood [1,18]. Another method to quantify exposure is the reported individual perceptions of his/her food environment [19]. While the use of objective measures is an important way to evaluate exposure to the food environment, residents’ perceptions on the availability of food retailers may differ from what is objectively available [20]. The assumption that what is available (i.e., food retailers) is also used by individuals might not always be true, especially in urban areas featuring many available options to obtain food; therefore, a current gap in food environment research is the predominant lack of information on the use of facilities by the participants [21]. The perception of residents on the presence and distance to food retailers was indicated as an important methodological alternative to understand the influence of the environment on food behavior [22].

The aim of this study was to test the associations between parental reports of their children’s use of food retailers and parental perceived travel time to food retailers with school children’s dietary patterns. We also aimed to examine effect modification by income levels.

## 2. Methods

### 2.1. Study Design, Sampling and Data Collection

This cross-sectional study is part of a wider research project funded by the Brazilian National Council for Scientific and Technological Development (CNPq). The project aimed to analyze the tendency and prevalence of obesity and its associated factors among primary and secondary school children from seven to 14 years old from Florianópolis, southern Brazil.

Details on the sampling and design of the project were reported by previous studies [23,24]. In summary, a clustered sampling process was performed where the schools were divided into strata according to the administrative regions of the municipality of Florianópolis (central, north, east, and south island and mainland), and the type of school (public or private). In total, 30 schools (19 public and 11 private) were randomly selected from the strata.

Data were collected from September 2012 to June 2013 by trained researchers. Anthropometric measurements of school children (weight and height) were collected according to a protocol defined by the World Health Organization (WHO) [25], which uses reference curves based on the recommendations of Lohman, Roche, and Martorell (1988) [26]. A questionnaire with information on sociodemographic characteristics and a food acquisition profile was completed by the parents or guardians of the students.

The research protocol was approved by the Ethics Committee of the Federal University of Santa Catarina (UFSC) (ethical code: 120341/2012), in accordance with the standards established by the Resolution No. 466/2012 of the Brazilian National Health Council. The study was conducted in accordance with the Declaration of Helsinki, and only school children whose parents or guardians signed the informed consent term participated in the study.

### 2.2. Measures

#### 2.2.1. Parents’ Reports of Children’s Usage of Food Retailers and Perceived Distance to Food Retailers

We asked parents how often (never, weekly, twice a month, monthly, or rarely), in the past six months, their children used restaurants and fast-food restaurants. Fast-food restaurants may refer to both commonly known fast-food chains that sell burger-based meals and also to small local-owned food retailers that sell traditional baked and deep-fried snacks. Parents were also asked whether or not they buy foods to cook at home in the supermarket. Parents answered how far (minutes of walking distance) restaurants, fast-food restaurants, and supermarkets were located from their home. Response options in minutes were “one to five”; “six to 10”; “11 to 15”; “16 to 20”, or “more than 20 min”.

Based on the data distribution, we categorized the reported frequency of use of restaurants and fast-food restaurants in the past six months into two categories (“did not use” or “used”). The use of supermarkets was originally asked in two categories (“did not use” or “used”). Parents’ perceived travel time was also categorized into three categories: less than 10 min, 10 to 20 min, and more than 20 min of walking distance from their home.

#### 2.2.2. Dietary Patterns

Food consumption information was collected using a qualitative food frequency questionnaire named QUADA (Portuguese acronym for “previous day food questionnaire”), an instrument developed and validated by Assis et al. [27], designed to assess the previous day food consumption of school children. QUADA presented higher kappa values for foods such as rice (k = 0.92), beans (k = 0.78), pasta (k = 0.81), vegetable soup (k = 0.82), and fruit (k = 0.78), and lower values for foods such as sweets (k = 0.38) and sweetened beverages (k = 0.47). QUADA is composed of 21 commonly consumed foods, with the same 21 foods presented chronologically over six meals (breakfast, morning snack, lunch, afternoon snack, dinner, and evening snack) [27]. The children completed the questionnaires at school. Guided by a trained researcher assistant, the children were asked to indicate which of the 21 available foods they had eaten during each meal on the previous day.

We derived dietary patterns performing factor analysis via the principal component method. The sum of all foods consumed by the children in the previous day were entered into the factor analysis which was performed using a polychoric correlation matrix—the most suitable for our food consumption data [28]. The obtained factors were then transformed by orthogonal rotation varimax. To test whether our dietary data were suitable for factor analysis, we used the Bartlett’s sphericity and Kayser–Meyer–Olkin (KMO) tests [29]. The criteria we used to retain the factors were an eigenvalue greater than 1, the scree plot charts, and the interpretability of the factors. Food groups were retained in the corresponding factor if they had a factor loading greater than or equal to ±0.3.

### 2.3. Covariates

Sociodemographic characteristics were obtained from a questionnaire answered by the parents including information on children’s sex (male or female), age (continuous), type of school (public or private), mother’s self-reported weight and height (used to calculate mother’s body mass index (BMI; continuous)), mother’s education, and household income. Response options for mother’s education were no formal education, incomplete secondary education, complete secondary education (equivalent to nine years of education), incomplete high school, complete high school (equivalent to 12 years of education), incomplete higher education, and complete higher education (university degree or higher). The variable maternal education was categorized into “complete secondary education”, “complete high school”, and “complete higher education”. Household income was continuously assessed via a questionnaire referring to the sum of the monthly net income of all household residents with a paid job. We split the continuous variable of income into tertiles to test effect modification by income levels. School children’s nutritional status was determined in terms of anthropometric measures; weight and height of school children were objectively measured and used to calculate the BMI. The school children’s BMI was then classified into “normal weight” and “overweight” following the WHO age and sex-standardized cut-offs [30]. The percentage of underweight children was lower than 2%; therefore, these children were incorporated into the normal-weight category.

### 2.4. Statistical Analyses

All the school children with complete food consumption data were kept in the analysis, resulting in an analytical sample of 2484 participants. Assuming that incomplete values were missing at random, we applied multiple imputation to all variables, except the outcome, using the predictive mean-matching method [31]. The percentage of missing values in our dataset ranged from 0.9% on children’s BMI to 30.0% on the variable “perceived travel time to fast-food restaurants”; therefore, we imputed 40 datasets as recommended by Rubin [32] and Bodner [33]. For descriptive statistics, non-imputed data were used, and, for the regression models, we used pooled results from the 40 imputed datasets. We also present the regression models using non-imputed data as a sensitivity analysis (Tables S1 and S2, Supplementary Materials).

To test the association between parental perceived travel time to supermarkets, restaurants, and fast-food restaurants, we built six different models to test the associations between the three “perceived travel time” variables and reported usage of supermarkets, restaurants, and fast-food restaurants with dietary patterns. We tested for clustering at the school and at the grade levels across all models. Firstly, a random intercept for school was added, followed by a random intercept for grade. Using two-log likelihood tests, we found these two levels to be significant and, therefore, performed linear mixed-models analysis with participants (first level) clustered within grade (second level) and school (third level). We created product terms for the variables of use and income levels, as well as for the variable of perceived walking distance and income levels. To test effect modification, we added these product terms to the respective models. All models were a priori adjusted for age, children’s nutritional status, mother’s BMI and educational attainment, household income, and type of school. Questionnaire data were entered by two independent researchers and processed by Software EpiData^®^ 3.2 (Epi Info Centers for Disease Control and Prevention, Atlanta, Georgia, United States). Descriptive and multilevel linear regression analyses, as well as multiple imputations, were performed using Stata^®^ 13.0 (Statistical Software for Professionals, College Station, TX, United States).

## 3. Results

Participants characteristics are described in Table 1. The mean age of school children was 10.4 years old, while the majority were girls (56.5%) and were classified as normal weight (66.8%). Most mothers had up to 12 years of education (46.1%); the mean mothers’ BMI was 25.2 kg/m^2^. A large majority of parents reported the use of supermarkets in the past six months (95.7%); restaurants and fast-food restaurants were reported to be used by 65.6% and 64.1% of parents, respectively. Most participants reported to live 10 min or less from a supermarket (40.6%) and from a fast-food restaurant (42.1%), whereas 40.2% reported to live more than 20 min away from a restaurant.

The factor loadings for each food item in the corresponding dietary pattern are presented in Table 2. Five dietary patterns were derived from factor analysis: The first dietary pattern, named “Fast Food”, was composed of French fries, soft drinks, pizza, packaged snacks, treats, and pasta, and it explained 12.5% of the data variance. The second dietary pattern, named “Morning/Evening Meal”, was composed of cheese, milk, coffee, yogurt, and breads, and it explained 12.3% of data variance. These foods are usually consumed as breakfast or a morning snack, and, in the south of Brazil, a warm meal for dinner is commonly replaced by a meal composed of bread, cheese, milk, and coffee. The third dietary pattern, named “Traditional Brazilian”, was composed of rice, beans, meat, and leafy greens, and it explained 10.5% of the data variance. The fourth dietary pattern, named “Healthy/Fresh Foods”, was composed of juice, fruits, vegetables, leafy greens, soup, and fish, and it explained 9.9% of the data variance. The fifth dietary pattern, named “Bread and Chocolate Milk”, was composed of chocolate milk and bread, and it explained 6.2% of the data variance. The total data variance explained by the five dietary patterns was 51.4%.

Table 3 shows the association of parents’ reported use of food retailers and dietary patterns. We found that children whose parents reported using supermarkets had 0.19 higher score (95% confidence intervals (CIs): 0.00, 0.38) in the “Morning/Evening Meal” pattern. Children whose parents reported using a restaurant had 0.09 higher score (95% CIs: 0.01, 0.18) in the “Fast Food” pattern. Children whose parents reported using fast-food restaurants had 0.13 higher score in the “Fast Food” pattern (95% CIs: 0.05, 0.20), 0.08 higher score (95% CIs: 0.00, 0.16) in the “Bread and Chocolate Milk” pattern, and 0.09 lower score (95% CIs: −0.17, −0.02) in the “Morning/Evening Meal” pattern. Table 4 shows the association of parents’ perceived travel time to the supermarket, full-service restaurants, and fast-food restaurants with dietary patterns. School children whose parents reported living more than 20 min away from a restaurant had 0.12 lower score (95% CIs: −0.21, −0.02) in the “Fast Food” pattern. School children whose parents reported living more than 20 min away from fast-food restaurants had 0.18 lower score (95% CIs: −0.27, −0.08) in the “Fast Food” pattern. No association was found for parents’ perceived travel time to supermarkets and dietary patterns. Parents’ perceived travel time to any of the food retailers analyzed was not associated with the other four dietary patterns. We did not find any effect modification effects by income levels.

Tables S1 and S2 (Supplementary Materials) show results from the sensitivity analysis where we performed the linear regression analyses using non-imputed data. In general, the results from sensitivity analyses were very similar to the main analyses where we used imputed data, with the exception of the association of use of fast-food restaurants and the “Morning/Evening Meal” pattern, which was significant in the main analysis β −0.09 (−0.17, −0.02) and became non-significant in the sensitivity analysis β −0.08 (−0.16, 0.00). All other significant associations found in the main analysis were also found with the non-imputed dataset. Other few associations that were not significant in the main analysis became significant with non-imputed data, for instance, the positive association between the use of supermarkets and the “Healthy/Fresh Foods” pattern. However, due to the higher chance of bias present in the complete case analysis (sensitivity analysis), we only interpret results from the analysis using imputed datasets.

## 4. Discussion

We explored the associations between parents’ reported use of food retailers by school children and parents’ perceived travel time to food retailers with school children’s dietary patterns. We found an association between the use of supermarkets and a higher score in the “Morning/Evening Meal” pattern. The use of full-service restaurants and fast-food restaurants was associated with a higher score in the “Fast Food” pattern. Higher perceived travel time to full-service restaurants and fast-food restaurants was associated with a lower score in the “Fast Food” pattern. We found no association between the use of food retailers and the “Traditional Brazilian” or the “Healthy/Fresh” foods pattern. No association was found either for perceived travel time to food retailers and the “Morning/Evening Meal”, the “Traditional Brazilian”, the “Healthy/Fresh Foods”, or the “Bread and Chocolate Milk” patterns. No effect modification was found by income levels.

The fact that no association was found for the use of and perceived distance to food retailers and the “Traditional Brazilian” and the “Healthy/Fresh Foods” patterns is surprising, as we expected, for instance, that the use of and greater perceived distance to fast-food restaurants would be inversely associated with both “Traditional Brazilian” and the “Healthy/Fresh Foods” patterns. The hypothesis would be that traditional meals and fresh foods would more likely be consumed at home, meaning fewer visits to fast food restaurants. As shown by previous Brazilian studies, eating out of home is associated with the consumption of unhealthy foods (e.g., soft drinks, salty snacks, pizza, and sweets) [34,35], while a higher frequency of meals prepared at home is associated with lower calorie intake from foods rich in solid fat and sugar [36]. Yet, it could also be that the lack of variation for the use of supermarkets (96% reported using it) might have undermined our ability to detect any significant associations, which would mean that, even if an association was present, we would not be able to detect it.

The use of full-service restaurants and fast-food restaurants, which is linked to the consumption of foods away from home, was associated with the dietary pattern composed of less healthy foods. Powel and Nguyen investigated the association of eating in full-service restaurants and fast-food restaurants and increased energy intake [37]. They found that eating in full-service restaurants was associated with higher daily energy intake and higher intake of sugar-sweetened beverages among American children and adolescents (2–19 years old). A Brazilian study conducted by Andrade et al. [17] identified three dietary patterns for out-of-home food consumption: traditional meal, typical Brazilian breakfast/tea, and ultra-processed food. The authors found that children and adolescents aged 10–19 years had higher adhesion to the ultra-processed food pattern than older individuals [17]. This may indicate that, even when eating in restaurants that offer healthy options for lunch and dinner, such as the commonly found buffet-by-weight restaurants in Brazil, children and adolescents would choose less healthy options. Indeed, taste preferences for energy-dense foods could influence the selection of less healthy foods in restaurants offering this type of food [38].

The association between use of fast-food restaurants and the first dietary pattern was expected, as fast-food consumption is largely related to unhealthy dietary patterns. Fast-food consumption is associated with a lower overall diet quality in American children aged 2–18 years old; children characterized as high consumers of fast food presented a higher intake of sugar-sweetened beverages and French fries, and a lower consumption of fruits and vegetables when compared with children characterized as low consumers [39]. A positive association between sugar-sweetened beverage consumption and the use of fast-food restaurants was also identified in Chinese adolescents [40]. We previously observed a positive association between the use of fast-food restaurants and the consumption of ultra-processed foods with the same characteristics of food groups found in the “Fast Food” pattern [24].

Despite the positive association of the use of full-service restaurants and fast-food restaurants and a higher score in the “Fast Food” pattern, the perception of living further away from these outlets seems to have a protective effect on children’s diet; higher perceived travel time to full-service restaurants and fast-food restaurants was negatively associated with the “Fast Food” pattern. Although the effect sizes found in our study are rather small, considering all individual and environmental factors that influence school children’s dietary behavior, we consider those to be relevant effect sizes. Similar to our findings, Cutumisu et al. [41] found a positive association between the exposure to fast-food restaurants around schools and unhealthier food consumption at lunchtime, suggesting that the environment that school children are exposed to has an influence in their diet. The relationship between distance from fast-food restaurants and diet was also found in a study from the United States (US). In this study, children (four to 14 years) who lived closer to fast-food restaurants were less likely to consume fruits and vegetables [42].

Some studies explored the perceived availability of fast-food restaurants and found a positive association with a higher consumption of unhealthy foods among children and adolescents [12,43]. The majority of studies on food environment research used objectively measured metrics; however, literature using this methodology is inconsistent [44]. It may be that perceived access measures could produce more consistent results [3]. In addition, individual perception of the food environment is also likely to influence behavior, and it may reflect how people experience and interact with their neighborhoods. Thus, measures of perceived access to the food environment might be an important tool for food environment research and should also be taken into account when trying to understand the relationship between food environment and eating behaviors [45].

Our results are subject to some limitations, such as the fact that QUADA assesses the frequency of intake rather than quantity. Regarding the use of food retailers, when asking parents how often their children use restaurants and fast-food restaurants, we did not ask if the children were accompanied by the parents or not. This could lead to a mistaken report, especially from parents of older children. However, we assume that parents would know when their children visited restaurants, even when the parents were not present. The social desirability bias could be considered, especially among the older children, which could feel discouraged to report the consumption of less healthy foods in the presence of their peers and researchers. A common limitation with the use of food questionnaires is memory bias. Nonetheless, QUADA was developed to minimize difficulties related to children’s assessment of portion size and to avoid lack of memory, since it accesses foods consumed in the previous day. In addition, during the administration of the questionnaire, researchers ensured that the students were situated in time and space while presenting the foods in the QUADA chart in a chronologic order. Although dietary intake from one day only may not be representative of the habitual individual intake, it is an advantage of our study that it included the coverage of different weekdays, including weekdays and Sundays [27]. We were also able to obtain a large sample size. This study is innovative for evaluating the information of both perceived travel time and the use of facilities, which is lacking in the current food environment research.

## 5. Conclusions

Our results indicate that, although the use of restaurants and fast-food restaurants may be associated with the “Fast Food” pattern, the perception of living further away from these food retailers might have a positive influence on school children’s diet. These findings highlight the importance of evaluating the perceived food environment and the use of food retailers, which can provide a complementary understanding of the relationship between the food environment and diet.

## Figures and Tables

**Table 1:** Descriptive characteristics of the participants. Florianópolis, Brazil, 2012–2013 (*n* = 2484).

Characteristics	Total Sample	
	***n***	**Mean (SD)**
**Age (years)**	2484	10.4 (2.2)
**Mother’s BMI (kg/m^2^)**	2264	25.2 (4.6)
	***n***	**%**
**Sex**		
Male	1162	43.5
Female	1322	56.5
**Household income (net per month)**		
Tertile 1 (31–482 USD)	712	31.9
Tertile 2 (483–917 USD)	725	34.1
Tertile 3 (942–24,465 USD)	694	34.0
**Mother’s education level**		
Complete secondary education	630	24.5
Complete high school	1058	46.1
Complete higher education	679	29.4
**Nutritional status**		
Normal weight	1645	66.8
Overweight	817	33.2
**Parents’ perceived travel time from home to supermarket**		
≤10 min	883	40.6
>10 to ≤20 min	659	29.3
>20 min	693	30.2
**Parents’ perceived travel time from home to restaurant**		
≤10 min	606	33.6
>10 to ≤20 min	461	26.2
>20 min	757	40.2
**Parents’ perceived travel time from home to fast food restaurant**		
≤10 min	688	42.1
>10 to ≤20 min	482	27.2
>20 min	570	30.8
**Parents’ reported own use of supermarkets**		
Did not use	91	4.3
Did use	2284	95.7
**Parents’ report on children’s use of restaurants**		
Children did not use	845	34.4
Children did use	1503	65.6
**Parents’ report on children’s use of fast-food restaurants**		
Children did not use	911	35.9
Children did use	1424	64.1

BMI = body mass index; USD = United States dollar.

**Table 2:** Factor loadings ^a^ of dietary patterns among children and adolescents investigated. Florianópolis, Brazil, 2012–2013 (*n* = 2484).

Food Groups	Fast Food	Morning/Evening Meal	Traditional Brazilian	Healthy/Fresh Foods	Bread/Chocolate Milk
Breads	−0.2524	**0.3852**	0.0137	0.0675	**0.5599**
Chocolate milk	0.2368	−0.1373	−0.0048	0.0920	**0.6870**
Milk	0.0265	**0.7989**	0.1080	0.1052	−0.0357
Coffee	−0.0725	**0.7703**	0.0532	−0.1155	−0.1773
Yoghurt	0.2298	**0.6560**	0.0152	0.1256	0.0211
Cheese	0.0717	**0.8033**	−0.0451	0.1036	0.1933
Rice	−0.0465	0.0083	**0.9251**	0.0034	−0.0054
Soft drinks	**0.6898**	0.0264	0.1005	−0.2326	0.1400
Treats	**0.5396**	0.0187	0.0409	−0.0470	0.2058
Chips	**0.6252**	0.0627	−0.0345	0.0348	0.0625
French fries	**0.7282**	0.0798	0.0825	0.0314	−0.0862
Pizza	**0.6668**	0.0192	−0.2274	−0.0019	−0.0763
Fruits	0.0233	0.1199	0.0325	**0.6938**	−0.0403
Beans	0.0548	0.0455	**0.8172**	0.1228	−0.0273
Pasta	**0.3310**	0.1290	−0.2188	0.1168	0.1829
Fish	0.2855	0.0324	0.1986	**0.3465**	−0.2197
Meat	0.1271	0.1456	**0.5172**	−0.1101	0.2787
Juice	−0.0996	−0.0058	−0.0162	**0.7213**	0.0693
Leafy greens	−0.1343	0.1110	**0.3807**	**0.5015**	0.1376
Soup	0.2081	0.0882	−0.1974	**0.4682**	−0.3393
Vegetables	−0.1405	0.1198	0.2450	**0.5748**	0.2395
% explained variation	12.5	12.3	10.5	9.9	6.2
% of accumulated explained variance	12.5	24.8	35.3	45.2	51.4

^a^ Highlighted values indicate a high correlation between the food group and the dietary pattern.

**Table 3:** Coefficients and 95% confidence intervals (95% CIs) as derived from multilevel linear regression analyses indicating the associations between parents’ reported use of supermarkets and their children’s usage of restaurant and fast-food restaurants with dietary patterns of school children. Florianópolis, Brazil, 2012–2013 (*n* = 2484).

	Fast Food β (95% CIs)	Morning/Evening Meal β (95% CIs)	Traditional Brazilian β (95% CIs)	Healthy/Fresh Foods β (95% CIs)	Bread/Chocolate Milk β (95% CIs)
**Parents’ reported own use of supermarkets**					
Did not use	1	1	1	1	1
Did use	−0.05 (−0.25, 0.15)	**0.19 (0.00, 0.38)**	−0.04 (−0.24, 0.15)	0.12 (−0.06, 0.31)	−0.03 (−0.23, 0.17)
**Parents’ report on children’s use of restaurants**					
Children did not use	1	1	1	1	1
Children did use use	**0.09 (0.01, 0.18)**	−0.03 (−0.12, 0.05)	−0.05 (−0.13, 0.04)	0.06 (−0.02, 0.14)	0.03 (−0.05, 0.12)
**Parents’ report on children’s use of fast-food restaurants**					
Children did not use	1	1	1	1	1
Children did use	**0.13 (0.05, 0.20)**	**−0.09 (−0.17, −0.02)**	−0.01 (−0.08, 0.07)	−0.03 (−0.10, 0.04)	**0.08 (0.00, 0.16)**

All models were adjusted for age, type of school, nutritional status, maternal BMI, household income, and maternal education. Results in bold are statistically significant.

**Table 4:** Coefficients and 95% confidence intervals (95% CIs) as derived from multilevel linear regression analyses indicating the associations between parents’ perceived travel time to supermarkets, restaurants, and fast-food restaurants with dietary patterns of school children in Florianópolis, Brazil, 2012–2013 (*n* = 2484).

	Fast Food β (95% CIs)	Morning/Evening Meal β (95% CIs)	Traditional Brazilian β (95% CIs)	Healthy/Fresh Foods β (95% CIs)	Bread/Chocolate Milk β (95% CIs)
**Parents’ perceived travel time from home to:**					
**Supermarkets**					
≤10 min	1	1	1	1	1
>10 to ≤20 min	−0.04 (−0.13, 0.05)	−0.05 (−0.14, 0.05)	−0.01 (−0.10, 0.08)	−0.04 (−0.13, 0.04)	−0.07 (−0.17, 0.02)
>20 min	−0.03 (−0.12, 0.06)	−0.01 (−0.11, 0.08)	0.08 (−0.01, 0.17)	0.06 (−0.03, 0.15)	−0.09 (−0.18, 0.01)
**Restaurants**					
≤10 min	1	1	1	1	1
>10 to ≤20 min	0.00 (−0.10, 0.11)	0.04 (−0.06, 0.15)	−0.01 (−0.13, 0.10)	−0.01 (−0.11, 0.09)	−0.04 (−0.15, 0.08)
>20 min	**−0.12 (−0.21, −0.02)**	0.06 (−0.04, 0.15)	0.04 (−0.07, 0.14)	0.03 (−0.07, 0.12)	−0.04 (−0.14, 0.06)
**Fast-food restaurants**					
≤10 min	1	1	1	1	1
>10 to ≤20 min	−0.05 (−0.15, 0.05)	0.04 (−0.06, 0.14)	−0.05 (−0.15, 0.05)	0.06 (−0.04, 0.15)	−0.03 (−0.14, 0.07)
>20 min	**−0.18 (−0.27, −0.08)**	0.05 (−0.04, 0.16)	0.00 (−0.10, 0.10)	0.06 (−0.04, 0.15)	−0.06 (−0.16, 0.04)

All models were adjusted for age, type of school, nutritional status, maternal BMI, household income, and maternal education. Results in bold are statistically significant.

## Supplementary Materials

The following are available online at https://www.mdpi.com/1660-4601/16/5/824/s1: Table S1: Coefficients and 95% confidence intervals (95% CIs) as derived from multilevel linear regression analyses indicating the associations between parents’ reported use of supermarkets and their children’s usage of restaurant and fast-food restaurants with dietary patterns of school children. Florianópolis, Brazil, 2012–2013 (*n* = 2484); Table S2: Coefficients and 95% confidence intervals (95% CIs) as derived from multilevel linear regression analyses indicating the associations between parents’ perceived travel time to supermarkets, restaurants, and fast-food restaurants with dietary patterns of school children in Florianópolis, Brazil, 2012–2013 (*n* = 2484).
