# Supplementary material for: Parental Perceived Travel Time to and Reported Use of Food Retailers in Association with School Children’s Dietary Patterns
Source: Int J Environ Res Public Health. 2019 Mar 7;16(5):824. doi: 10.3390/ijerph16050824 (PMC6427110; doi:10.3390/ijerph16050824)
Supplement: Supplementary file 1 [file ijerph-16-00824-s001.pdf]

Sensitivity analyses with non-imputed data

Table S1. Coefficients and 95% confidence intervals (95%CI) as derived from multilevel linear regression analyses indicating the associations between parent's report use of supermarkets and their children's usage of restaurant and fast food restaurants with dietary patterns of schoolchildren. Florianópolis, Brazil, 2012–2013 (*n* = 2484).

	Fast Food β (95%CI)	Morning Snack β (95%CI)	Traditional Brazilian β (95%CI)	Healthy/Fresh Foods β (95%CI)	Bread/Chocolate Milk β (95%CI)
**Parent's reported own use of supermarkets (*n* = 1942)**					
Did not use	1	1	1	1	1
Did use	-0.04 (-0.24; 0.16)	**0.26 (0.05; 0.46)**	-0.04 (-0.25; 0.17)	**0.24 (0.04; 0.43)**	-0.09 (-0.12; 0.30)
**Parent's report on children's use of restaurants (*n* = 1925)**					
Children did not use	1	1	1	1	1
Children did use use	**0.12 (0.02; 0.21)**	-0.02 (-0.11; 0.06)	-0.04 (-0.13; 0.06)	0.05 (-0.03; 0.14)	0.03 (-0.06; 0.12)
**Parent's report on children's use of fast food restaurants (*n* = 1917)**					
Children did not use	1	1	1	1	1
Children did use	**0.14 (0.06; 0.23)**	-0.08 (-0.16; 0.00)	-0.01 (-0.09; 0.08)	-0.04 (-0.12; 0.04)	0.07 (-0.01; 0.16)

All models were adjusted for age, type of school, nutritional status, maternal BMI, household income, and maternal education. Results in bold are statistically significant.

Table S2. Coefficients and 95% confidence intervals (95%CI) as derived from multilevel linear regression analyses indicating the associations between parents' perceived travel time to supermarkets, restaurants and fast food restaurants with dietary patterns of schoolchildren in Florianópolis, Brazil, 2012–2013 (*n* = 2484).

	Fast Food<br>β (95%CI)	Morning Snack<br>β (95%CI)	Traditional Brazilian<br>β (95%CI)	Healthy/Fresh Foods<br>β (95%CI)	Bread/Chocolate Milk<br>β (95%CI)
**Parent's perceived travel time to:**					
**Supermarkets (*n* = 1831)**					
≤10 min	1	1	1	1	1
>10 to ≤20 min	-0.03 (-0.13; 0.07)	**-0.11 (-0.21; -0.01)**	-0.02 (-0.12; 0.08)	-0.06 (-0.16; 0.03)	**-0.11 (-0.21; -0.00)**
>20 min	-0.07 (-0.17; 0.03)	-0.02 (-0.11; 0.08)	0.06 (-0.04; 0.17)	0.06 (-0.03; 0.16)	-0.12 (-0.23; -0.02)
**Restaurants (*n* = 1507)**					
≤10 min	1	1	1	1	1
>10 to ≤20 min	0.04 (-0.08; 0.15)	0.01 (-0.11; 0.14)	0.00 (-0.12; 0.12)	0.02 (-0.09; 0.14)	-0.05 (-0.18; 0.07)
>20 min	**-0.14 (-0.24; -0.04)**	0.06 (-0.04; 0.17)	0.02 (-0.09; 0.13)	0.02 (-0.08; 0.13)	-0.02 (-0.13; 0.09)
**Fast food restaurants (*n* = 1437)**					
≤10 min	1	1	1	1	1
>10 to ≤20 min	-0.02 (-0.14; 0.08)	0.04 (-0.07; 0.16)	-0.08 (-0.20; 0.04)	0.10 (-0.01; 0.21)	-0.03 (-0.15; 0.09)
>20 min	**-0.18 (-0.29; -0.08)**	0.05 (-0.06; 0.16)	0.01 (-0.11; 0.12)	0.06 (-0.05; 0.16)	-0.07 (-0.19; 0.04)

All models were adjusted for age, type of school, nutritional status, maternal BMI, household income, and maternal education. Results in bold are statistically significant.
